# Annual Meeting of the American Veterinary College Alumni Association

**Published:** 1895-04

**Authors:** J. B. Hopper

**Affiliations:** Secretary


### ANNUAL MEETING OF THE AMERICAN VETERINARY COLLEGE ALUMNI ASSOCIATION.

THE Alumni Association of the American Veterinary College held their annual meeting in the College Hall, on Monday, March 25th, at 2 P.M. President Hanson called the meeting to order, after which Secretary Hopper called the roll. Some forty of the members responded to their names.

The report of the Executive Committee was then received, and the following recommendations of the Committee were approved: That the annual meeting shall hereafter be held at 3 P.M. on commencement day; that the Alumni Association should ask of the Board of Trustees a larger representation than they now accord.

Some eight deaths were reported by the Secretary as having occurred during the past year, as follows: Drs. John S. Saunders, Massachusetts, Class of '76; C. B. Michener, District of Columbia, Class of '76; E. W. Rowland, Wisconsin, Class of '82; J. C. Jackson, New York, Class of '87; P. F. Kiernan, Missouri, Class of '87; Robert L. Leis, New Jersey, Class of '89; Frederick D. Greeley, Pennsylvania, Class of '89; George B. Burchsted, New York, Class of '91. Suitable resolutions were adopted and ordered to be engrossed upon the minutes of the Association, and a copy of the same to be sent to the journals.

The Committee on Revision of Constitution and By-laws reported progress, and asked for more time to consider the many needed changes.

The election of officers for the ensuing year resulted in the choice of Drs. E. B. Ackerman, Class of '91, *President*; Herbert Neher, Class of '87, *Vice-President;* Martin J. Dair, Class of '95, *Secretary;* F. R. Hanson, Class of '95, *Treasurer;* and Bryce Mars, Class of '94, *Librarian.*

J. B. HOPPER,
Secretary.

